# Case report: electrical storm during induced hypothermia in a patient with early repolarization

**DOI:** 10.1186/s12872-017-0711-2

**Published:** 2017-11-15

**Authors:** Patrick Badertscher, Michael Kuehne, Beat Schaer, Christian Sticherling, Stefan Osswald, Tobias Reichlin

**Affiliations:** 1 Cardiovascular Research Institute Basel (CRIB), University Hospital Basel, Basel, Switzerland; 2 Department of Cardiology, University Hospital Basel, Petersgraben 4, 4031 Basel, CH Switzerland

**Keywords:** Early repolarization, Sudden cardiac death, Ventricular fibrillation, Electrical storm, Hypothermia, Antiarrhythmic drugs

## Abstract

**Background:**

Population based studies showed an association of early repolarization in the electrocardiogram (ECG) and a higher rate of sudden cardiac death presumably due to ventricular fibrillation. The triggers for ventricular fibrillation in patients with early repolarization are not fully understood.

**Case presentation:**

We describe the case of a young patient with a survived ventricular fibrillation arrest while asleep followed by multiple episodes of recurrent ventricular fibrillation. The admission ECG showed an early repolarization pattern with substantial J-point elevation in most of the ECG-leads. After initiation of a hypothermia protocol, the patient developed an electrical storm with multiple ventricular fibrillation episodes requiring multiple cardioversions. Intravenous isoproterenol infusion successfully suppressed the malignant arrhythmia.

**Conclusion:**

Hypothermia appears proarrhythmic in patients with early repolarization and may trigger ventricular fibrillation. This knowledge is particularly important when initiating temperature management protocols in patients after a survived cardiac arrest. During the acute phase of an early repolarization associated electrical storm, isoproterenol is the most effective treatment suppressing the ventricular fibrillation-inducing premature ventricular complexes at higher heart rates.

## Background

Population based studies showed an association of early repolarization (ER) in the electrocardiogram (ECG) and a higher rate of sudden cardiac death presumably due to ventricular fibrillation (VF) [1, 2]. The triggers for VF in patients with ER are not fully understood [3]. Based on this case we discuss the possible link between hypothermia and recurrent VF episodes in the context of ER.

## Case presentation

**Fig. 1:**
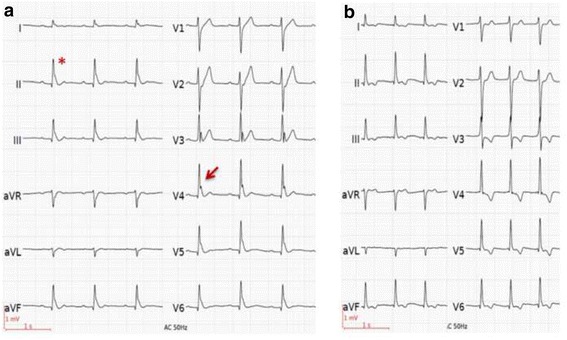
ECG at presentation (**a**) and during isoproterenol infusion (**b**). Panel A shows the electrocardiogram (ECG) at presentation showing major QRS-T abnormality with substantial J-point elevation in most of the ECG-leads and notching of the terminal part of the QRS complex in the lateral leads (arrow) and slurring of the terminal part of the QRS complex in the inferior leads (*). Panel B shows near-normalization of QRS morphology during isoproterenol infusion. The notch in the lateral leads disappeared and the slur in the inferior leads was markedly attenuated. New T wave inversions are present, probably attributed to cardiac memory

**Fig. 2:**
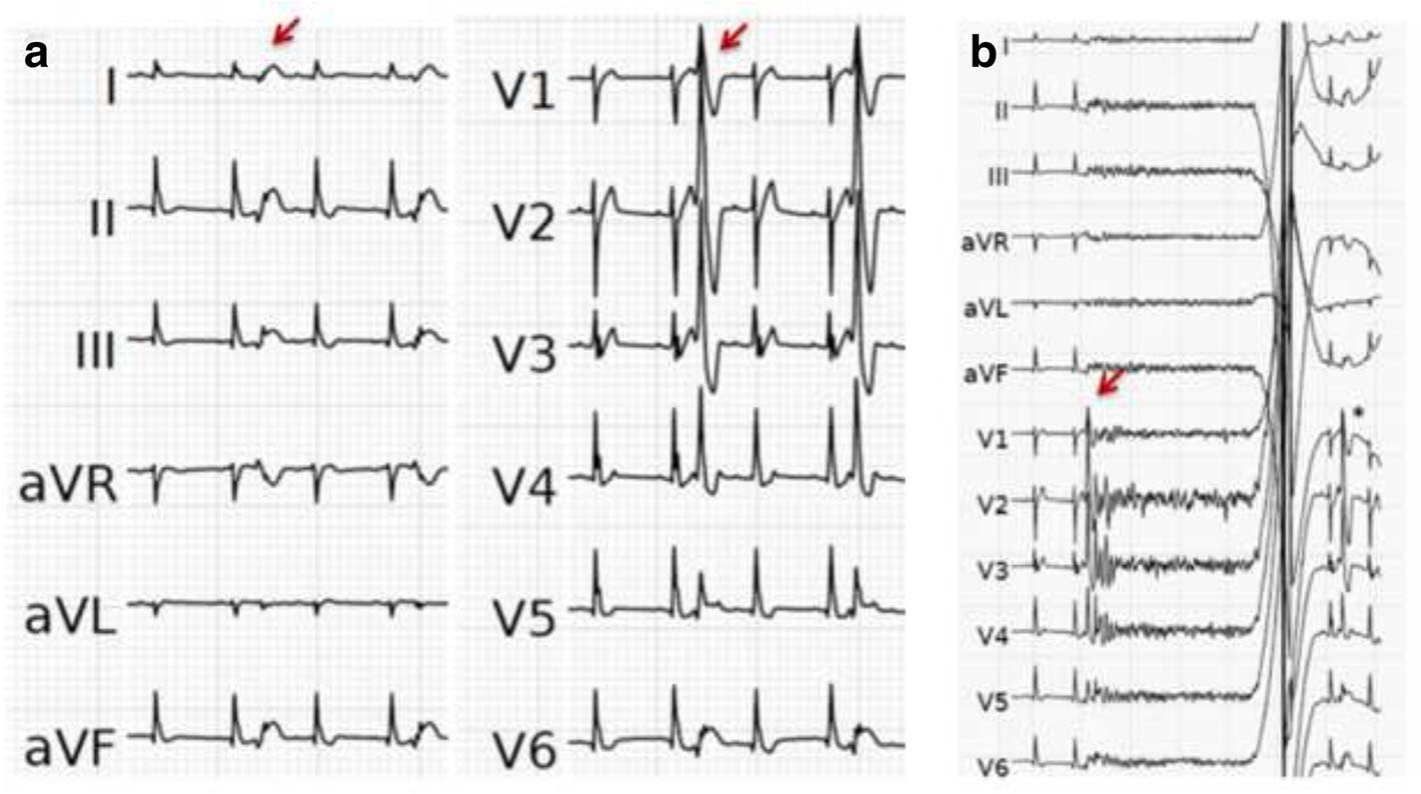
Episodes of early coupled PVCs (**a**) and PVC-induced Ventricular fibrillation (**b**). Panel A shows the early coupled PVC’s (arrow) occurring during the descending part of the T-wave, probably originating from the left anterior fascicle (right bundle branch block, inferior axis). Panel B shows one episode of PVC-induced VF (initiating PVC marked with arrow). Following defibrillation, the a next short-coupled PVC reoccurres immediately (*)

**Fig. 3:**
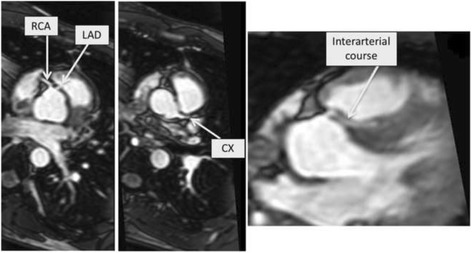
Cardiovascular magnetic resonance imaging showing interaterial course of left anterior descending artery. Cardiovascular magnetic resonance imaging showing the course of the anomalous left anterior descending coronary artery (LAD) arising from a separate ostium of the right sinus of Valsalva with a potentially malignant interarterial course, in which the LAD passes between the aorta and pulmonary trunk. The left circumflex (LCX) artery is located in its usual expected position at the left sinus of Valsalva

A 31-year-old man suffered an out-of-hospital cardiac arrest during the night while asleep. His girlfriend started CPR immediately. Upon arrival of the emergency medical team, the patient was found in VF and defibrillation could restore spontaneous circulation. He had no significant past medical history and was taking no medications. Family’s medical history revealed that the patient’s sister had implanted a pacemaker due to sick-sinus syndrome, at the age of 22 years. Plasma electrolyte levels, including potassium and calcium, were within normal ranges. Toxicological screening was negative. The ECG at presentation showed sinus rhythm and an ER pattern with substantial J-point elevation in most of the ECG-leads with notching of the terminal part of the QRS complex in the lateral leads and slurring in the inferior leads (Fig. 1). Coronary angiography revealed an anomalous origin of the left anterior descending (LAD) coronary artery arising from a separate ostium of the right sinus of Valsalva without any other concomitant congenital anomaly. In the absence of neurological recovery or purposeful movements, a targeted temperature management protocol with a goal temperature of 36 °C was started in the intensive care unit [4]. Three hours later, the patient began to experience very frequent episodes of spontaneous VF. The episodes of VF were induced by short-coupled monomorphic premature ventricular complexes (PVC) falling into the vulnerable descending part of the T-wave (Fig. 2). The PVCs showed a right bundle block morphology in V1 and a right-inferior axis in the limb leads, suggesting an origin in the left anterior fascicle. The patient was shocked for more than 10 VF episodes, but the PVC kept reoccurring, sometimes just a few beats after cardioversion (Fig. 2b). The patient’s condition was finally stabilized by isoproterenol infusion, which successfully suppressed the PVC’s at higher heart rates in sinus rhythm. During the course of the isoproterenol infusion, a reduction of the ER pattern on the 12-lead ECG was observed (Fig. 1b). Compared to the ECG at presentation (Fig. 1a) the notch in the lateral leads disappeared and the slur in the inferior leads was markedly attenuated. Remarkably, new T wave inversions were present, possibly attributed to cardiac memory. Two days later, the patient was extubated and showed a good neurologic recovery. Cardiac magnetic resonance (CMR) imaging demonstrated normal cardiac structure and function and no late gadolinium enhancement. Regarding the anomalous origin of the LAD from the opposite sinus CMR revealed an interarterial course, in which the LAD passes between the aorta and pulmonary trunk. The left circumflex (LCX) artery was located in its usual expected position at the left sinus of Valsalva (Fig. 3). A dual chamber ICD was implanted allowing for continued atrial pacing. A myocardial perfusion imaging with bicycle exercise stress testing was performed four weeks after the hospitalization and showed no signs of ischemia. The patient has not experienced any further arrhythmias during the first two months of follow-up.

## Discussion

ER is common, occurring in 1% to 5% of the general population [1]. It is more common in young adults (particularly men) and athletes. The ECG pattern is defined as the presence of a J-point elevation ≥1 mm in ≥2 contiguous inferior and/or lateral leads with or without ST-elevation, either as a QRS slurring (an abrupt change in the slope of the last deflection) or notching (a low-frequency deflection at the end of the QRS complex) [5]. These criteria are fulfilled in our patient as illustrated in Fig. 1a. ER syndrome (ERS) is diagnosed in the presence of this ECG pattern and resuscitation of otherwise unexplained VF or polymorphic VT [5].

Historically ER was considered benign. Population based studies by Tikkanen et al. [1] then showed a higher rate of SCD among patients with ER, case-control studies by Haïssaguerre et al. [2] found an increased prevalence of ER in patients with idiopathic VF (31%–42%) compared with healthy control subjects (5%–13%). This was true for J-point elevations in the inferior leads and leads I to aVL, but not in leads V4–V6, where the frequency was similar. Importantly, subjects with ER and idiopathic VF were more likely to have sustained a cardiac arrest during sleep, which was also the case in our patient. Haïssaguerre et al. [2] noted an increase in the amplitude of ER prior to an arrhythmic period. This finding was consistent with a preliminary analysis [6] of four patients with ER and idiopathic electrical storm, in which an unique electrocardiographic signature was found. A baseline ER pattern with dramatic but transient accentuation of J waves across the precordial and limb leads before the development of the electrical storm, which was precipitated by relatively short-coupled PVCs, was observed in all four patients. The same electrocardiographic signature was seen in our patient: the ER pattern was most pronounced immediately before the electrical storm, with subsequent ECG recordings after the electrical storm demonstrating a progressive decrease in J-wave height and slurring, respectively notching (Fig. 1a vs. b).

With regard to risk stratification, Antzelevitch et al [3] described three subtypes of ER with a varying risk profile: in type 1, the ER pattern is limited to the lateral precordial leads. It is typically seen in healthy male athletes and has the lowest risk of malignant arrhythmias. Type 2 shows ER in the inferior and inferolateral leads and is associated with a greater risk of malignant arrhythmias and type 3 shows ER pattern in all ECG leads and has the highest risk of malignant arrhythmias and electrical storms. The ER pattern of our patient was most likely compatible with type 2. In addition to the location, a J-point elevation of >0.2 mV was previously shown to be linked to a significant risk of death from cardiac arrhythmias [1]. And also distinct T-wave features have been used for the risk stratification in ER patients [7].

Different triggers leading to an electrical storm can be discussed. Recent reports point to an association between ER and the development of VF in the setting of hypothermia [8–10]. Current guidelines recommend a targeted temperature management protocol to prevent neurological damage following a cardiac arrest. In our patient, a target temperature of 36 °C was used. Electrical storm started and exacerbated after initiation of cooling and no arrhythmias occurred after it was stopped, suggesting a temporal relationship between hypothermia and increased frequency of ventricular arrhythmias in our patient. Although Brugada syndrome and ER differ in many aspects, a possible relationship to temperature is suspected in both entities, as fever appeared to be proarrhythmic in Brugada patients [11].

The finding of an LAD originating from the right sinus of Valsalva is extremely rare (0.03%) [12]. In general, this subtype of an anomalous origination of a coronary artery from the opposite sinus (ACAOS) is associated with congenital heart disease, e.g. tetralogy of Fallot. The anatomical variant with an interarterial course of the LAD between the ascending aorta and the pulmonary trunk is considered high risk for myocardial ischemia or sudden death as well [13]. The mechanism of sudden death is believed to be due to transient occlusion of the LAD caused by an increase in blood flow through the aorta and the pulmonary artery, resulting in kinking or pinching of the artery [13]. In our case, an interarterial course was present. However, before admission, the patient was asymptomatic without any complaints during daily activity and regular exercise and the VF arrest occurred while sleeping, making ACAOS and resulting myocardial ischemia a very unlikely cause of his VF arrest. Owing to a lack of clear guideline recommendations, the management of patients with ACAOS remains fraught with uncertainty. Of note, there are no controlled studies that have evaluated the outcome of surgical repair in asymptomatic individuals. We decided that further risk stratification with myocardial perfusion imaging stress testing (MPI) was warranted. The MPI was performed four weeks after the hospitalization and showed no signs of ischemia.

Different therapeutic management options for electrical storms in ERS patients can be discussed. For the acute phase, beta-blockers, verapamil and lidocaine/mexiletine are generally ineffective, while amiodarone may be partially effective (3/10 patients) [14]. The most effective strategy in the acute phase of electrical storms however seems the use of isoproterenol infusion or pacing at rapid heart rates to suppress the PVC’s [14]. In addition, Isoproterenol is thought to be effective probably by boosting L-type calcium channel current and thus decreasing electrical gradient [3]. The efficacy of isoproterenol infusion has additionally been reported in a case report of an electrical storm occurring in a patient with Brugada syndrome [15]. For the long-term prevention of recurrent VF in ERS patients, quinidine is the drug of choice [14]. Catheter ablation for idiopathic VF targets the short coupled PVC triggers. In experienced hands, it can have a good long-term success in preventing VF recurrence [16]. However, presence of the PVC’s during the procedure is required for mapping and successful ablation and recurrent VF episodes may be induced during ablation due to triggered automaticity. Therefore, quinidine is often chosen as the first line option for long-term suppression and catheter ablation used in patients with recurrent electrical storms in ERS patients. Importantly, due to the limited data with regard to sample size and follow-up duration [14, 16], neither successful catheter ablation nor quinidine should be considered alternatives to the implantation of an ICD for secondary prevention of SCD. In summary, ER is associated with idiopathic VF and hypothermia appears proarrhythmic in patients with ER and idiopathic VF. It remains uncertain whether additional avoidable triggers of arrhythmogenesis exist in patients with ER. Interventions shown to be effective are isoproterenol and quinidine. Further research of the genetics and mechanisms involved are required.

## Conclusion

Intensive care physicians should be aware of hypothermia as a possible trigger of electrical storm in patients with ERS. This knowledge is particularly important when initiating temperature management protocols in patients after a survived cardiac arrest. During the acute phase of an electrical storm, isoproterenol is the most effective treatment suppressing the VF-inducing PVCs at higher heart rates. For the long-term management, both catheter ablation of the PVC triggers and quinidine have been used successfully to reduce or even prevent recurrent VF episodes.

## Abbreviations

CMR: Cardiac magnetic resonance
ECG: Electrocardiogram
ER: Early repolarization
ERS: Early repolarization syndrome
ICD: Implantable cardioverter-defibrillator
LAD: Left anterior descending
PVC: Premature ventricular complexes
VF: Ventricular fibrillation
